# 3D-printed microplate inserts for long term high-resolution imaging of live brain organoids

**DOI:** 10.1186/s42490-021-00049-5

**Published:** 2021-04-01

**Authors:** Mariana Oksdath Mansilla, Camilo Salazar-Hernandez, Sally L. Perrin, Kaitlin G. Scheer, Gökhan Cildir, John Toubia, Kristyna Sedivakova, Melinda N. Tea, Sakthi Lenin, Elise Ponthier, Erica C. F. Yeo, Vinay Tergaonkar, Santosh Poonnoose, Rebecca J. Ormsby, Stuart M. Pitson, Michael P. Brown, Lisa M. Ebert, Guillermo A. Gomez

**Affiliations:** 1 Centre for Cancer Biology, SA Pathology and University of South Australia, Adelaide, SA 5000 Australia; 2 ACRF Cancer Genomics Facility, Centre for Cancer Biology, SA Pathology and University of South Australia, Frome Road, Adelaide, SA 5000 Australia; 3 Institute of Molecular and Cell Biology (IMCB), Agency for Science, Technology and Research (A-STAR), Singapore, Singapore; 4 Department of Pathology, Yong Loo Lin School of Medicine, National University of Singapore, Singapore, Singapore; 5 Department of Neurosurgery, Flinders Medical Centre, Adelaide, SA 5042 Australia; 6 Flinders Health & Medical Research Institute, College of Medicine & Public Health, Flinders University, Adelaide, SA 5042 Australia; 7 School of Medicine, University of Adelaide, Adelaide, SA 5000 Australia; 8 Cancer Clinical Trials Unit, Royal Adelaide Hospital, Adelaide, SA 5000 Australia

**Keywords:** Brain organoids, Live-imaging, Fluorescence microscopy, Glioblastoma

## Abstract

**Background:**

Organoids are a reliable model used in the study of human brain development and under pathological conditions. However, current methods for brain organoid culture generate tissues that range from 0.5 to 2 mm of size, which need to be constantly agitated to allow proper oxygenation. The culture conditions are, therefore, not suitable for whole-brain organoid live imaging, required to study developmental processes and disease progression within physiologically relevant time frames (i.e. days, weeks, months).

**Results:**

Here we designed 3D-printed microplate inserts adaptable to standard 24 multi-well plates, which allow the growth of multiple organoids in pre-defined and fixed XYZ coordinates. This innovation facilitates high-resolution imaging of whole-cerebral organoids, allowing precise assessment of organoid growth and morphology, as well as cell tracking within the organoids, over long periods. We applied this technology to track neocortex development through neuronal progenitors in brain organoids, as well as the movement of patient-derived glioblastoma stem cells within healthy brain organoids.

**Conclusions:**

This new bioengineering platform constitutes a significant advance that permits long term detailed analysis of whole-brain organoids using multimodal inverted fluorescence microscopy.

**Supplementary Information:**

The online version contains supplementary material available at 10.1186/s42490-021-00049-5.

## Background

Brain organoids [1] have emerged as promising models to better understand brain development and the mechanisms that contribute to the pathology of different brain diseases including Alzheimer’s Disease, Parkinson’s Disease and brain cancer [2–4]. Cerebral organoids are generated from human embryonic (hESC) or induced pluripotent (iPSC) stem cells, which, after directed differentiation, lead to the emergence of different cell populations that self-organise to form tissue structures resembling the developing (foetal) human cerebral cortex [1, 5–7]. Since the original publication of this method [1], various protocols have been reported, including those suitable for the growth of brain region-specific organoids [1, 7–12]. It is now possible to induce systematic differentiation of stem cells into dorsal and ventral cortices [1, 13], hindbrain, midbrain [1, 7–12], cerebellum [14] and subcortical structures such as the hypothalamus [11] and hippocampus [15]. Furthermore, neurons within brain organoids produce electrical activity [12, 16] and can support functional neuromuscular junctions [17]. These results indicate that brain organoids provide an important in vitro platform technology with which to mimic the cellular composition as well as the cytoarchitecture and function of brain tissue [1, 18].

Brain organoids can also be generated from patient-derived tissue samples, which – in many cases – reproduce critical features of the brain disease [11, 19–22], constituting a significant technological advantage for the study of these diseases. For example, this technology has revealed how impairment of neural progenitor cells (NPCs) found in Zika virus infection at various stages of human embryogenesis contribute to microcephaly [11] as well as used to recapitulate different Alzheimer’s Disease pathologies, such as amyloid aggregation, Tau hyper-phosphorylation and endosome abnormalities [23]. Patient-derived brain organoids could also be used to produce patient-specific tissue for tissue regeneration after insults such as tumour biopsies, neurodegenerative disease and neurological trauma [24, 25].

Brain organoids generated from patient-derived tissue have also attracted significant interest in the clinical setting, particularly in brain cancer, including glioblastoma, and thus constitute a powerful pre-clinical model for the development of personalised treatment strategies [3]. In this regard, glioblastoma-brain organoid models reproduce key characteristics of the brain cancer microenvironment found in glioblastoma patients, including hypoxic gradients [20], pathological features [26], cellular diversity and gene expression [22]. Furthermore, glioblastoma-brain organoid models can be used to assess patient response to current standard of care treatments in addition to novel immunotherapies [22, 26, 27]. Patient-derived brain organoids have the potential to predict patient prognosis rapidly, assess treatment response and improve diagnosis. Taken together, increasing the clinical management of aggressive cancers such as glioblastoma [28] and avoiding the ‘trial and error’ approaches of current clinical practice, which can be costly, ineffective and present limited benefits to patients.

However, a significant limitation of brain organoid technology that hinders its application to personalised medicine is the lack of a methodology that allows temporal (e.g. days, weeks, months) live high-resolution imaging of multiple organoids in a multi-well plate. Such a method would allow evaluation of treatments using patient-derived organoids, while concomitantly determining the effect of these treatments on various disease parameters. This is particularly important in brain cancer, where current approaches generally focus on cancer cell proliferation and neglect fundamental malignant processes such as invasion in which cancer cells interact with both the cellular and non-cellular microenvironment [3, 28, 29]. In this report, we describe the use of 3D-printed microplate inserts that solve this problem, by enabling the culture of multiple brain organoids in specified XYZ coordinates, thus facilitating high-resolution imaging using fluorescence microscopy.

## Results

### 3D-printed microplate inserts to culture multiple brain organoids

**Fig. 1:**
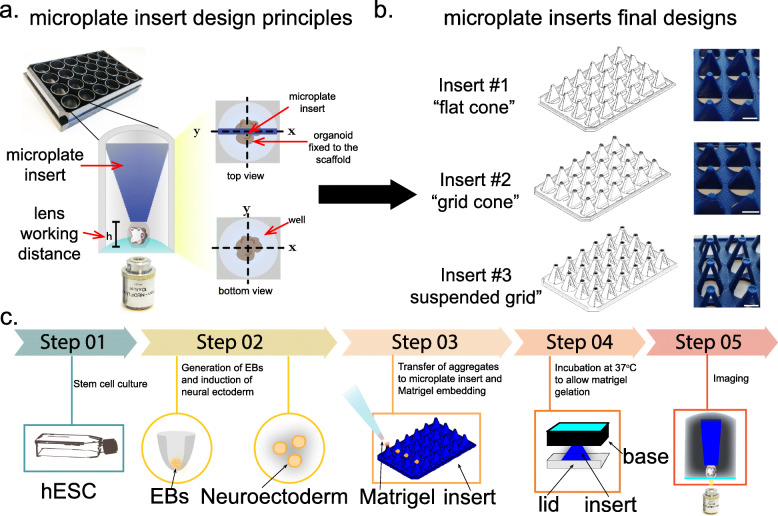
Microplate inserts for brain organoid culture. **a** Design principle for microplate inserts for brain organoid culture in fixed XYZ positions compatible with high-resolution imaging. **b** Final design of the three types of microplate inserts. Scale bar = 10 mm. **c** Pipeline for growth of cerebral organoids using microplate inserts

To facilitate the culture of multiple brain organoids in specified XYZ positions within a microplate, we designed a series of 3D-printed microplate inserts for standard 24-well microplates (Fig. 1a) that permit:

1. the culture of multiple brain organoids (30–60 day-old) using glass bottom microplates suitable for imaging applications.
2. the confinement of the brain organoid to the axial (Z) position to facilitate whole-organoid live imaging by:
  i. maintaining the brain organoid within the working distance of the objective (2.5 mm).
  ii. minimising the effect (contact, pressure) that the presence of the inserts may exert on brain organoids, as this confinement is compatible with > 30 day-old organoid dimensions (~ 1 mm average diameter, [30]).
3. the fixation of the XY position of the organoid within individual wells to facilitate automated imaging acquisition and post-acquisition analysis as the organoid develops.

We anticipate that by controlling these three parameters, the engineered microplate inserts will be ideal for high-resolution, whole-organoid live imaging, and be suitable for automated microscopy that is required for relatively fast and systematic readouts like those employed in drug screening.

As a result of our design requirements, we were able to produce three different microplate inserts that satisfy the stated design principles (Fig. 1b). For simplicity, we named these microplate inserts as flat-cone (insert #1), grid-cone (insert #2), and suspended-grid (insert #3). A shared design element of each insert is the ability to secure the organoid tissue close to the glass bottom of the microplate within the objective working distance (Fig. 1a).

A fine mesh (“grid”) present on the tip of inserts #2 and #3, is a unique feature that minimises the potential interaction surface that might occur between the organoid and the insert. In the case of insert #3, a hollow space present on the side opposite to the grid aids with media exchange and oxygenation of the organoids [30].

We employed a Raise 3D Pro printer for printing the final microplates (Fig. 1b; see also Methods) and cultured brain organoids following standard protocols developed by Lancaster and Knoblich with small modifications [1, 30] (Fig. 1c). For Step 1 of this procedure, we used hESC H9 [31], H9-GFP [32] or a mixture of both in a ratio 100 H9: 1 H9-GFP (“chimera organoids”) to generate embryoid bodies (EBs) in low-attachment U-shaped 96 well plates (Fig. 1c). In Step 2, EBs were matured to form neuroectodermal aggregates. In Step 3, we placed the microplate inserts facing upwards over a multi-well plate lid, which permits the transfer and positioning of the aggregates on the tip of the inserts. In Step 4, a droplet of Matrigel was added on top of each organoid and then the plate base (without the lid and facing downwards) was placed on top of the insert. Following Matrigel gelation by incubation at 37 °C, the multi-well plate containing the inserts was returned to the normal orientation before the addition of culture medium for further organoid culture, differentiation and imaging (Step 5, Fig. 1c).

### Microplate inserts allow long-term monitoring and individual assessment of brain organoid growth using automated fluorescence microscopy

**Fig. 2:**
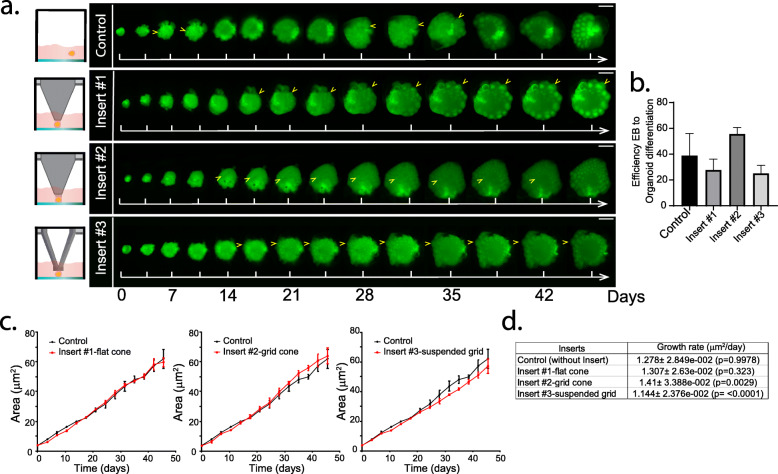
Time course of live brain organoid imaging using microplate inserts. **a** Images were taken every ~ 3.5 days using an InCell Analyzer microscope using a 2.1x objective. Control refers to organoids grown without microplate inserts. Organoids grown with inserts #1 to #3 maintain their orientation and produce characteristic cortical structures (yellow arrows). Panels were made using cropped images (same size for all time points and conditions) placed on a black background. Image’s intensity levels were contracted (same histogram contraction applied to panels from all time points across all conditions) from their original 16-bit range to 8-bit RGB range for Figure preparation in Adobe Illustrator. Scale Bar = 1 mm. **b** Percentage of EBs that successfully matured to brain organoids under different conditions was determined by fluorescence microscopy at day 50. Successfully grown organoids were identified by an increase in size over time and identification of characteristic cortical structures. Data are means ± SEM for three independent experiments. **c** Organoid 2D projected area (Area) vs time plots for brain organoid cultures in the presence or absence of microplate inserts. Data points correspond to mean values for three independent experiments (*n* = 3), and for each experiment at least 3 organoids per condition were analysed. **d** Results of linear regression analysis (Area = initial organoid size + Growth rate * Days) of plots in **c**. The table shows the *p* values for the comparison of organoid growth rates between the control and microplate insert groups. Non-Significant (n.s) and Significant (*, *p* < 0.05)

In order to test the advantages of using microplate inserts for culture and imaging of mature brain organoids, we performed long term imaging of individual brain organoids starting immediately after Matrigel embedding (Day 0) for 50 continuous days, which has not previously been reported. For this, organoids grown in multi-well plates with and without inserts were imaged every 3.5 days (84 h) for 50 days using wide field automated fluorescence microscopy (2x objective, InCell Analyzer 2200, Cytiva). Figure 2a shows still images from different time points taken for organoids grown with and without microplate inserts. A key advantage of using microplate inserts is that the organoids are grown in a fixed position, preserving their orientation and permitting the tracking of individual features within the organoid (yellow arrows) in contrast to the control organoids grown without inserts (Fig. 2a and Supplementary Movie 1).

We then assessed the effect of the different microplate inserts on organoid formation. For this, we determined the efficiency of EB to organoid differentiation by counting the proportion of organoids that successfully formed and exhibited characteristic cortical structures (yellow arrows Fig. 2a) by day 50. The different microplate inserts were compared to the control condition without insert. Figure 2b shows the results of 3 independent experiments with 24 organoids (i.e. one multi-well plate) per experiment. The differentiation efficiency was ~ 40% in all experimental and control conditions. We observed less variability (measured as standard error of mean) for conditions using microplate inserts compared to the control conditions without inserts. Moreover, cultures using microplate insert #2 showed a trend (although non-significant) of yielding a larger proportion of organoids when compared to all the other conditions (with or without insert, Fig. 2b). Together, these results suggest that the use of microplate inserts and the restriction of organoid movement within the well, did not alter the capacity of EBs to differentiate to organoids.

We then evaluated the effect of microplate inserts on brain organoid growth rates. For this we analysed the time series of acquired images (Fig. 2a) by automatically segmenting organoids based on their fluorescence intensity and measuring the organoid z-projected area (Scripts for image segmentation are provided in the Supplementary Information). For successfully grown organoids (Fig. 2b), the organoid projected area was plotted as a function of time (Fig. 2c) and organoid growth rates and organoid initial projected area determined by linear regression (Fig. 2d and Supplementary Table 1).

Firstly, we confirmed that the organoid projected area at time 0 (i.e. immediately after embedding in Matrigel) was similar either in the presence or absence of microplate inserts (Fig. 2c, Supplementary Table 1). This suggested that the presence of the insert does not exert pressure on the organoids which might significantly alter their shape and projected area [33, 34]. This also reduced the potential for differences in organoid growth rates arising from different initial EB size.

Analysis of growth rates at later time points revealed that cultures grown with microplate insert #1 grew at rates comparable to control conditions without an insert (Fig. 2c and d). Organoids grown using microplate insert #2 exhibited a significantly higher growth rate, compared to the controls after 30 days while organoids grown using microplate insert #3 resulted in a significantly slower growth rate (Fig. 2c, d). Of note, although we found slight differences in organoid growth rates in different conditions, the size of the organoids was not significantly different between inserts and controls at any time point of our analysis. For example, at day 50, calculations using data from our linear regression analysis of growth rates, determined organoid area to be 66.3 ± 2.2, 66.7 ± 2.0, 71.1 ± 2.6 and 59.8 ± 1.8 μm^2^ for control (no insert), insert 1, insert 2 and insert 3, respectively. These values correspond to 0.6 ± 6%, 7 ± 7% and − 10 ± 6% differences in size between organoids grown using inserts 1–3 compared to organoids grown without inserts, respectively. One-way ANOVA statistical analysis of the variation in size was determined to be non-significant indicating that the presence of the inserts did not lead to altered 2D organoid areas as would have been expected by an increase in pressure or a highly forced confinement of the organoids due to the presence of the microplate inserts. Importantly, we performed staining for cleaved-caspase 3 in organoid sections to assess apoptotic cell death. Although we found cleaved caspase 3 positive cells principally in the periphery of cortical structures, the distribution of these apoptotic cells was not dramatically different between organoids using the different microplate inserts (Supplementary Figure 1).

Altogether, these results indicate that the presence of the microplate inserts, which facilitate long-term fluorescence microscopy imaging of live brain organoids, do not interfere with organoid growth when compared to the reference methods of organoid production [1, 30, 33].

### Human brain organoids grown using microplate inserts maintain human cerebral cortical organisation

**Fig. 3:**
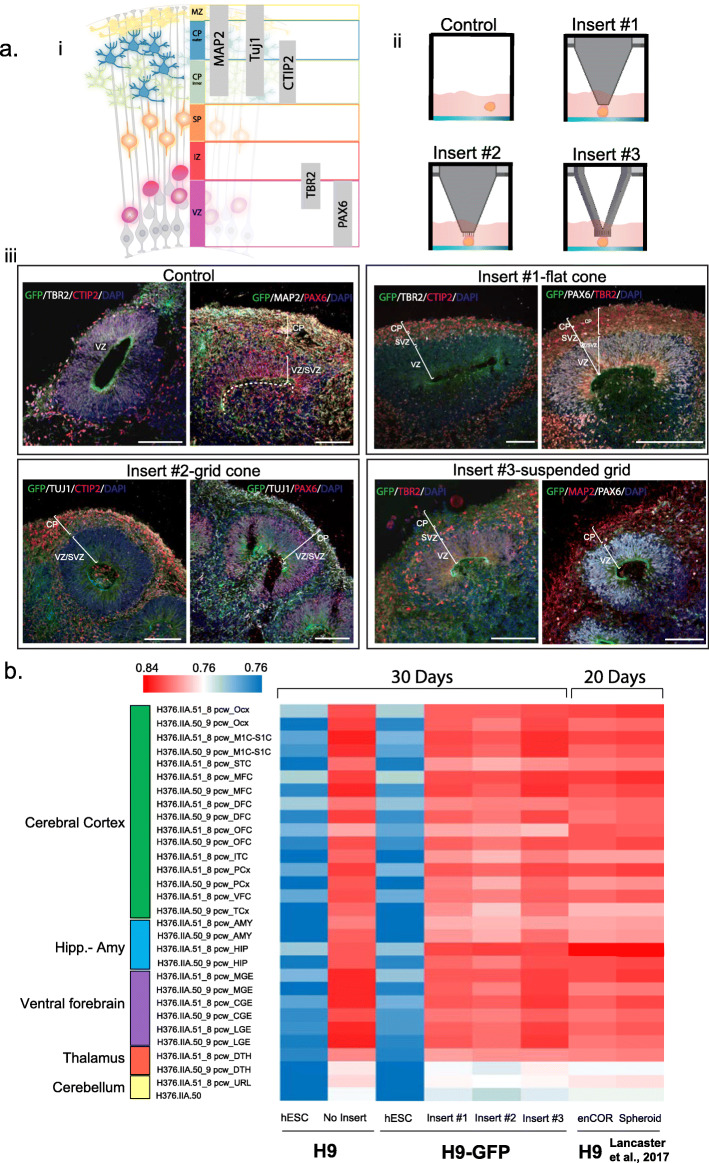
Brain organoids grown using microplate inserts show cerebral cortex identity. **a** i. Schematic showing the distribution of neuronal differentiation markers in the brain cortex. ii. Schematic representation of brain organoid cultures using the different microplate inserts. Control refers to the condition without insert. iii. Representative sections of cerebral organoids grown using the microplate inserts. Each tissue contains neural progenitors (PAX6^+^), intermediate progenitors (TBR2^+^), and neurons (CTIP2^+^, Tuj1^+^, MAP^+^), with no significant differences observed in the cytoarchitecture of the cerebral cortex of the brain organoids grown under the different experimental conditions (i.e. with or without microplate inserts). Panels were made with cropped images (same size for all panels shown). Image’s intensity levels were contracted at the same extent for all individual channels, across all panels shown to increase visibility and maximize compatibility between original 16-bit range -microscope image output- and 8-bit range (RGB) for Figure preparation in Adobe Illustrator. Scale Bar = 100 μm **b** Heatmap of Spearman correlation analysis of gene expression between H9 and H9-GFP hESC, brain organoids (grown with and without microplate inserts), previously published organoid RNA-seq data [33] and BrainSpan database https://www.brainspan.org/static/download.html. Hipp-Amy: Hippocampus-Amygdala

We then used immunofluorescence to analyse in more detail the cytoarchitecture (Fig. 3a, i) of 30 day-old brain organoids grown in the presence or absence of microplate inserts (Fig. 3a, ii). For this, brain organoids were embedded and frozen in OCT, sectioned and immune-labelled with different markers (Fig. 3a, iii). Staining with neural progenitor marker (Pax6) revealed a typical ventricular region within organoids, which was comparable between all conditions analysed (Fig. 3a, iii, control and inserts #1-#3). Tbr2-positive intermediate progenitors cells (IPCs), which are neuronal progenitors that divide away from the ventricular surface [34], were present in the proliferative region of the cerebral organoids grown in different conditions, an observation that is in agreement with previously published reports for brain organoids [1, 7]. Furthermore, staining for CTIP2, MAP2 and TUJ1 confirmed that brain organoids grown using microplate inserts contain neurons that have properly migrated within the preplate and which should therefore subsequently mature in a cortical plate structure [35]. Thus, cortical zones in brain organoids generated using microplate inserts display typical progenitor zone organisation as previously shown for brain organoids grown using standard methods [1, 30, 33].

To further validate these results, we performed RNA sequencing (RNA-seq) experiments to determine the gene expression profile of 30 day-old organoids grown using microplate inserts for comparison with gene expression signatures reported for different brain regions early in development [36, 37] and with brain organoids grown using reference methods [1]. To achieve this, we prepared single cell suspensions of 30 day-old brain organoids derived from H9 and H9-GFP cells and performed RNA-seq. Gene expression profiles from brain organoids cultured using microplate inserts and data previously reported [33] were then compared to those derived from different brain regions (cerebral cortex, hippocampus, amygdala, ventral forebrain, thalamus and cerebellum) collected from the BrainSpan database (https://www.brainspan.org/static/download.html [36, 37]). Comparison of gene expression patterns for markers from different brain regions was performed by calculating Spearman correlation coefficients between gene expression values derived from the organoids and those reported in the BrainSpan data base [33]. As a control, we also included non-differentiated hESCs. In general, we found gene expression profiles of H9-GFP organoids (inserts #1–3), but not h9-GFP hESC, correlated well with the profiles from different brain regions, particularly the cerebral cortex, hippocampus/amygdala and ventral forebrain. These patterns agree with data obtained for organoids grown using standard protocols (20 day-old enCOR, Lancaster dataset, Fig. 3b). We also observed that the RNA expression profiles of 30 day-old organoids grown in our experimental conditions correlate less with thalamus and cerebellum, similar to the findings of Lancaster et al. using 20 day-old enCOR organoids [33]. Comparing gene expression, we observed a high correlation between conditions using microplate inserts #3, no insert H9 (Control), and 20-day-old enCOR (Fig. 3b). These results show that the expression profiles of organoids using microplate inserts are comparable to those generated using standard protocols [1, 30, 33].

### Use of microplate inserts to track cerebral cortex development process and monitor tumour growth and invasion within brain organoids

After determining that microplate inserts allow the proper growth and development of brain organoids, we wanted to test whether this technology enables whole organoid live imaging and the tracking of individual cells within the tissue using high-resolution microscopy. For this we focused on two biological processes that are important in brain development and disease: (i) neuronal progenitor behaviour including interkinetic nuclear migration (INM) and cell division orientation, which are key processes that occur during neocortex expansion [38, 39] and (ii) glioma stem cell migration, invasion and proliferation, central steps in glioblastoma development and progression [29].

**Fig. 4:**
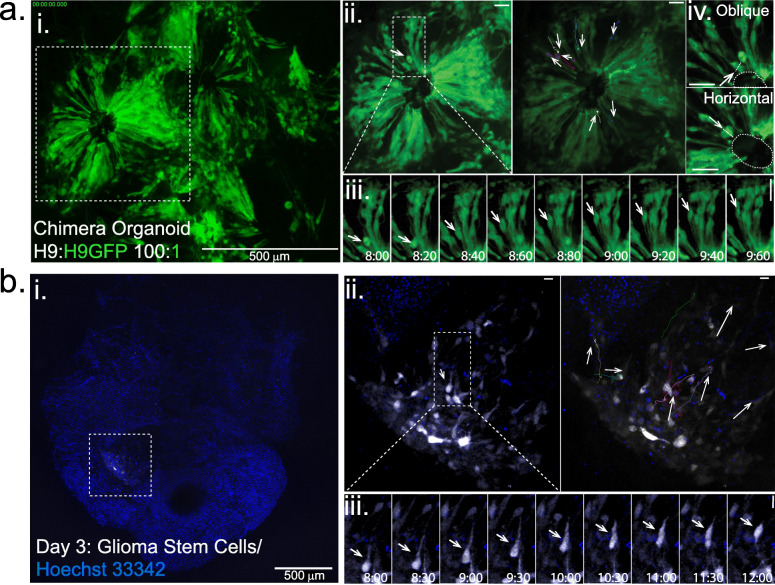
Live brain organoid imaging. **a** Neuronal progenitor behaviour in brain organoids (see also Supplementary Movie 2). i. Whole brain organoid projection. ii. Magnified view of a brain organoid cortical area. iii. Neuronal movement tracking in a brain organoid region and still images showing INM. iv. Examples of neuronal progenitor oblique and horizontal cell division. Scale bars are 500 μm (i) and 50 μm (ii-iv). **b** Visualisation of GSC tumour growth and invasion within healthy brain organoids using confocal microscopy (see also Supplementary Movie 3). i. Time-lapse (h) of cerebral organoids injected with patient-derived glioma stem cells (PD-GSC). ii. Magnified view of region highlighted in i (left) with individual cell trajectories (right). iii. Still images showing GSC movement within the brain organoid. In **a** and **b**, Panels were made with cropped images (same size for all time points and conditions). Image’s intensity levels were contracted (same extent for all time points across all conditions but different across different channels) from their original 16-bit range to 8-bit RGB for Figure preparation in Adobe Illustrator

For the analysis of INM, and to increase the contrast of individual cells, we prepared “chimera organoids”, which result from the differentiation of EBs produced using a mixture of H9 and H9-GFP in a 100:1 ratio (Fig. 4a, i). After 30 days’ growth using microplate insert #3, these organoid chimeras showed a visible mosaic of cells expressing GFP, which correspond to neuronal progenitors and neurons at different stages of differentiation (Fig. 4a, i-ii). At this point in differentiation, we performed confocal laser scanning for live cell imaging to analyse cell movement within the organoid (Fig. 4b, iii, Supplementary Movie 2). Analysis of spindle orientation of dividing cells throughout the movie revealed that the majority of cells dividing at the apical surface predominately exhibited horizontal and oblique spindle orientation (Fig. 4a, iv), as is expected from the division of neuronal progenitors [38]. Thus, live-imaging of intact whole cerebral organoids using the microplate inserts captures key characteristics of neuronal progenitor cells’ behaviour. Note that it was not possible to generate comparable data for organoids grown without inserts, due to an inability to keep the organoids in focus and track the same area over time, thus highlighting the importance of our new approach.

We then analysed glioma stem cell behaviour within brain organoids using live fluorescence microscopy imaging (Fig. 4b, i, Supplementary Movie 3). For this, we used patient-derived glioma stem cells (PD-GSC), which we fluorescently labelled using DNA-encoded fluorescent reporter RFP using lentivirus transduction [40, 41]. PD-GSC stably expressing RFP were injected directly into H9-GFP derived 30 day-old brain organoids grown using microplate insert #2. For injection, the microplate inserts with attached organoids were taken out of the microplate and inverted to allow injection of PD-GSC cells on top of the organoid. After injection, the microplate insert was returned to the microplate and cultured overnight before initiating live imaging to monitor the growth and invasion of the injected tumour cells into the surrounding healthy brain organoid (Fig. 4b, ii). Using live imaging, we were able to track individual cancer cells invading the surrounding healthy brain organoid, a process that showed some degree of collective behaviour (Fig. 4b, iii, Supplementary Movie 3). Thus, this assay permitted the assessment of PD-GSC behaviour within a physiologically relevant tissue context, which could have applications for the pre-clinical evaluation of potential anti-cancer therapies. Such assays have the potential to identify effective therapies that are efficient at targeting tumour heterogeneity and resistance to therapy [42–47], tumour invasion [26, 48], as well as drug-toxicity to the surrounding brain tissue [3].

## Discussion

Previously, different approaches have been taken to develop multi-well plate based microscopy assays for neural spheroids [49, 50] and brain organoids [51] and such approaches have been applied to brain cancer [48, 52]. Although useful, these methods have several disadvantages. Firstly, most of them are optimised for the culture of neurospheres [50], which are 3D aggregates of neurons without a brain tissue cytoarchitecture or very immature organoids (i.e. < 10 day-old [48]), that lack key features present in mature cerebral organoids (> 30 days old) such as the presence of distinct cortical brain regions [7, 53], electrical waves [12, 16] and presence of individual neuronal cell populations [54]. Unfortunately, 3D culture protocols, as well as brain organoids grown on chips [51], are not suitable for high-resolution imaging of > 30 day-old cultured organoids and are incapable of tracking individual cells within the organoid, due to their non-adherent nature and the agitation conditions required for culturing [11].

In addition, the design of these microplate inserts contrasts with previous efforts [55, 56] where human brain organoids were cultured confined to a semi 2D-dimensional compartment (150 μm thickness). Although such a sub-millimetre confinement [55–57] facilitates high-resolution imaging, this approach lacks utility for mature organoids which are large in size, thus reducing their pre-clinical applicability [22].

In this report, we present the development of new 3D-printed microplate inserts that allow the growth of brain organoids in standard multi-well plates and permit the scale-up of cerebral organoid culture. Development of 3D-microplate inserts allowed the direct observation of individual cells in whole live organoids to assess their growth, migration and proliferation within a three-dimensional and more physiologically relevant microenvironment. Moreover, immunofluorescence, RNAseq, and analysis of organoid growth revealed that organoids grown using microplate inserts are comparable to those generated using standard protocols [1, 30, 33].

Although cerebral organoids grown using the methods described here did not produce a significant number of astrocytes and oligodendrocytes [7, 58] extending the culture for longer time periods using the microplate inserts and the addition of PDGF-AA and IGF-1 from days 50–60 and T3 from days 60–70 [58] would produce more complex organoids which could then be analysed via whole-organoid live high resolution microscopy.

The ability to perform long-term high-resolution imaging of mature brain organoids, makes this technology more amenable to high-content analyses used for screening cellular responses to therapies, an important process for personalised phenotypic screening for brain cancer and other diseases.

We expect our whole organoid imaging method, along with deep imaging methods such as multiphoton, light sheet and single plane illumination microscopy [59–63] that enable single cell resolution deep into tissues, will significantly improve our understanding of the spatiotemporal relationships between different cell types in development and during disease progression.

## Conclusions

This new bioengineering platform based on the use of 3D printed micro-plate inserts that fit standard tissue culture microplates constitutes a significant advance in the culture and analysis of mature brain organoids by enabling long term detailed analysis using high-resolution multimodal inverted fluorescence microscopy to better understand spatiotemporal relationship between cells in the organoid.

## Methods

### 3D printing

3D drawings of microplate inserts were performed in AUTOCAD (Autodesk) and sliced for 3D printing using IdeaMaker (Raise3D) software. 3D printing was performed using a Pro2 Dual Extruder 3D Printer (Raise3D) using polylactic acid (PLA) filaments (Raise3D).

### Culture and maintenance of hESC

H9 [31] and H9 Cre-LoxP (H9-GFP, [32] were obtained from WiCell https://www.wicell.org/ and grown in feeder-independent conditions as described in [30]. Briefly, cells were cultured in growth factor reduced Matrigel (Corning, cat#356230)-coated T-25 flasks at 2.25 μg Matrigel/flask using mTeSR1 medium (Stem Cell Technologies, cat# 85870). ROCK inhibitor (Y-27632, Stem Cell Technologies, cat#72304) was used for cells when thawing and splitting only. After cells reached 60–70% confluence (~3 days of culture), cells were detached using EDTA 0.5 mM (Invitrogen, cat#15575020) in sterile D-PBS without calcium and magnesium (Gibco, cat#14200166) and plated again on fresh Matrigel-coated flasks. For the experiments shown here, cells were kept for no longer than ten passages.

### Generation of human brain organoids

We used the protocol by Lancaster and Knoblich [30] with some modifications for the use of microplate inserts. Briefly, 70–80% confluent hESC in T-25 flasks were incubated with Accutase (Sigma-Aldrich, cat# A6964) to produce a single-cell suspension in DMEM/F12 medium (Gibco, cat# 11330057) supplemented with Knockout Serum Replacement (Gibco, cat# 10828028), bFGF (4 ng/ml, Preprotech, cat# AF-100-18B) and ROCK inhibitor (100 μM). Cells (18,000 cells per well) were then seeded in a low attachment 96-well plate (Corning, cat#CLS7007), to form EBs. For chimera organoids 18,000 cells of a mixture 100 (H9):1(H9-GFP) was used.

EBs with smooth edges [30] were then transferred to a low-attachment 24-well plate (Corning, cat# CLS3473) to induce the formation of the neuroectoderm by feeding with DMEM/F12 supplemented with N2 (Gibco, cat# 17502048) and 1 μg/μl of Heparin (Sigma-Aldrich, cat#H3149). The neuroepithelial aggregates were fed every day for a week and then processed for embedding into regular Matrigel (Corning, cat#354234) droplets in the presence or absence of microplate inserts.

For conditions using microplate inserts, first, microplate inserts were sterilised by immersion in 80% ethanol followed by 30 min exposure to UV light within a biosafety cabinet. After this, EBs were added on top of the insert tips, by placing the microplate inserts –with the insert tips facing upwards– on a lid of a 24-well glass-bottom plate (CellVis, Cat#P24–1.5H-N). The neuroepithelial aggregates were then placed on the top of the micro-plate inserts using 1000 μL wide-bore pipette tips (Thermo Scientific, cat#2079G), and covered with 20 μL of regular Matrigel. Then, and in an inverted position, the base of the microplate was placed on top of the microplate inserts. To keep the neuroepithelial aggregates in place within the wells of the microplate, matrigel gelation was induced for 20 min at 37 °C in this orientation. After gelation, the whole assembly was inverted, and 400 μL neural induction media was added [30].

For conditions not using microplate inserts (Control), embedding and Matrigel gelation was performed as described in [30] and then Matrigel domes containing neuroepithelial aggregates were transferred to a 24-glass-bottom microplate (CellVis, Cat#P24–1.5H-N). Two days after Matrigel embedding, all cultures were fed with Improved Differentiation Medium without vitamin A [30], supplemented with 3 μM CHIR99021 (Sigma-Aldrich, cat#SML1046). Finally, organoid cultures were cultured in an incubator with constant agitation using an orbital shaker (Thermo Scientific, Cat#88881102).

### Cerebral organoid analysis and immunofluorescence staining

Organoids were fixed in 4% paraformaldehyde for 30 min at room temperature, washed with Dulbecco’s phosphate-buffered saline (D-PBS) and dehydrated with sucrose (Sigma-Aldrich, cat#84097) 30% over-night at 4 °C. Individual tissues were frozen by embedding in a solution of 10% sucrose and 7.5% gelatine (Sigma-Aldrich, cat#G1890) and submerged in 2-methylbutane (Sigma-Aldrich, cat#270342) at − 50 °C. The cerebral organoids were cut with using cryostat (Leica, CM1850) generating 20 μm serial sections. After cutting, sections were kept at − 80 °C for long-term storage. For immunostaining, tissues sections were washed with DPBS to remove the remnants of gelatine, then permeabilised with a solution of 0.2% Triton X-100 (Sigma-Aldrich, cat#T9284) for 30 min at room temperature, and blocked with 5% BSA (Sigma-Aldrich, cat#A7030) for 1 h at room temperature. A mix containing different primary antibodies was prepared in blocking buffer (5% BSA in D-PBS). The antibody mixture (100 μL) was added to each glass slide and then covered with Parafilm and incubated overnight at 4 °C. Tissue sections were then washed with D-PBS and incubated with a secondary mix (prepared in 1% BSA) for 1 h at room temperature. Coverslips were mounted on the glass slides containing the tissue sections using ProLong™ Diamond Antifade Mountant (Molecular Probes, cat#P36961) for 1 h at room temperature and imaged on a Leica SP8 STED microscope (Leica Microsystems GmbH, Wetzlar, Germany).

### Antibodies

Primary antibodies were Pax6 (Mouse, BD Pharmigen, cat#561462, dilution 1:100), Pax6 (Rabbit, Abcam, cat#ab195045, dilution 1:300), Sox2 (Rabbit, Merck, cat#AB5603, dilution 1:200), TBR1 (Rabbit, Abcam, cat#ab31940, dilution 1:100), TBR2 (Rabbit, Abcam, cat#ab23345, dilution 1:100), TUBB3 (Mouse, BioLegend, cat#801201, dilution 1:300), MAP 2 (Mouse, Merck, cat#MAB3418, dilution 1:50), CTIP2 (Rat, Abcam, cat#ab18465, dilution 1:100) and Cleaved Caspase-3 (Asp175) Antibody (Cat# 9661, Cell Signaling Technology, dilution 1:100). Secondary antibodies were species-specific antibodies conjugated with AlexaFluor 488, 546, 594 or 647 (Invitrogen).

### Immunofluorescence imaging

All confocal images of organoid sections were taken using a TCS SP8 STED 3X microscope (Leica) using a HC PL APO CS2 20x/0.75 DRY objective lens at 0.1 μm/pixel. A white light laser (50% power) tuned at 488, 568 and 647 nm laser line using tuneable acoustic-optical beam splitter was used for excitation of GFP, Alexa 568 and Alexa 647 (5–20% intensity was used for excitation). A diode 405 laser was used for Hoechst 33342 (nuclei), with a 405 nm laser line (2% laser transmission was used for excitation). Emission was collected in the range 415–445 nm (Hoescht33342); 500–530 nm (GFP), 580–620 nm (Alexa 568) and 660-700 nm (Alexa 647) using spectral detectors (Leica) and Hybrid sensors (HyD, gain: 100, offset: 0). Z slices (600 nm optical section, 1 AU) were captured, but single optical slices at the plane of ventricular zones were shown (Fig. 3). Images were acquired and processed using Leica LAS X software equipped with Lighting module (Leica).

### PD-GSC culture

Freshly resected glioblastoma tumour tissue was obtained from the hospital operating theatre by the South Australian Neurological Tumour Bank and further processed to generate patient-derived glioma stem cells. For this, small pieces of the resected tumour tissue, or a slurry of tissue fragments generated by cavitron ultrasonic surgical aspirator (CUSA), were added to a GentleMACS C-tube (cat#130–093-237). Tissue dissociation was performed using the Miltenyi Human Tumour Dissociation kit (cat# 130–095-929), by adding enzymes to the tissue according to manufacturer’s instructions, placing the C-tube on the GentleMACS dissociator and running the recommended program. The tissue mixture was filtered with a 70 μm cell strainer and the resulting suspension centrifuged (5 min at 250 x *g*). The cell pellet was washed twice with DMEM (Gibco, cat#11995065) and an aliquot of cells was resuspended in StemPro NSC serum-free medium kit (Life Technologies; cat#A10509–01) supplemented with Glutamax (Gibco, cat#35050061) and transferred to a T-25 Matrigel-coated flasks (coating performed at 1/100 dilution in D-PBS). We used Matrigel-coated flasks for ongoing culture, and Accutase for passaging. Passaging was performed when cells reached 80–100% confluence.

Lentivirus infection of PD-GSC was performed for expression of RFP in PD-GSC. For lentivirus preparation, HEK293T cells were transfected using Lipofectamine 2000 (Invitrogen) with lentiviral plasmid CMV-RFP-T2A-Luciferase (System Biosciences) and packaging plasmids pLP1, pLP2 and pVSVG (Invitrogen), according to the manufacturer’s instructions. Viral supernatant was harvested after 96 h and added to primary PD-GSC cells with 2 μg/ml polybrene (Sigma) in StemPro NSC serum-free media (Life Technologies). Successful stable transduction of PD-GSC was confirmed by fluorescence microscopy.

### RNA sequencing

Single cell suspensions from organoids were generated by Accutase treatment followed by mechanical dissociation and passage through 70 μm cell strainer. Total RNA was isolated from one organoid per condition using TRIzol Reagent (Invitrogen, cat#15596026) according to the manufacturer’s instructions. Isolated RNA was further purified with Monarch RNA Cleanup Kit (NEB, cat#2040 L). RNA quality was validated in Agilent 2100 Bioanalyzer. All samples had RNA Integrity Number (RIN) > 8. RNA quantity was validated in Qubit 4 Fluorometer (Invitrogen). RNA-seq libraries were generated with KAPA standard RNA-seq HyperPrep kit (Kapa Biosystems) using 300 ng total RNA. Library quality was validated with the Agilent 2100 Bioanalyzer. PolyA+ enriched RNA-seq libraries from 16 human samples were multiplexed and sequenced on two separate runs using the Illumina NextSeq 500 platform and the stranded single end protocol with a read length of 75 bp. Sequenced reads derived from both runs were merged for each sample before further processing. Raw data, averaging 70.5 million reads per sample were analysed and quality checked using the FastQC program (http://www.bioinformatics.babraham.ac.uk/projects/fastqc). Reads were mapped against the human reference genome (hg19) using the STAR spliced alignment algorithm [64] (version 2.5.3a with default parameters and --chimSegmentMin 20, −-quantMode GeneCounts) returning an average unique alignment rate of 90%. The gene counts were TMM normalized using R (version 3.2.3) and edgeR [65] (version 3.3).

For comparison of our RNA-seq gene expression data to the Allen Human Brain Atlas transcriptome data set, we downloaded the gene expression values from FPKM (https://www.brainspan.org/static/download.html). We analysed the data provided for 8 and 9 post-conception weeks (pcw), filtered by the genes expressed in our samples, and then matched through gene symbol. In addition, we extended our correlation analysis with previously published RNA-seq data [33] from 20 and 60 day-old human brain organoids (Spheroid) and engineered cerebral organoids (enCOR), (GEO: GSE80538). Gene expression was compared between conditions using Spearman correlation analysis (MATLAB, see Supplementary Information).

### Live organoid imaging

For low resolution imaging, cerebral organoids grown on multi-well plates with different microplate inserts were imaged using the InCell Analyser 2200 imaging system (Cytiva) equipped with a 2X objective, 0.1 NA (Cytiva), a 5.5Mp scientific-grade 16-bit CMOS camera (Cytiva) and In Cell Analyzer Acquisition Software v4.5 28–9630-76UM AE (Cytiva). Images (2048 × 2048 pixels at 3 μm/pixel) were taken every 3.5 days, using 0.1 s exposure for each channel. The differential interference contrast (DIC) imaging modality was used to take brightfield images, while GFP signal was imaged using FITC excitation (475/28 nm) and emission filters (511.5/23 nm) and appropriate dichroic mirrors (QUAD1, Cytiva). Images from the entire plate were analysed using a custom-made Image J script included in the Supplementary Information. This script imports all images of the plate at all different time points into one stack and then uses GFP fluorescence for segmentation of the organoid, which is then used to measure GFP mean fluorescence and organoid size for different wells at each time point. After running the script, the results table was exported to Excel (Microsoft) and then to PRISM (GraphPad) for regression analysis and graphical representation. Data points correspond to mean values for three independent experiments (*n* = 3), with at least 3 organoids per condition analysed from each experiment.

Confocal images of whole organoid chimeras alone or injected with RFP-expressing PD-GSCs were taken using a TCS SP8 STED 3X microscope (Leica) using a NF 488 (Leica, for GFP) or SMD1 NF 405/470 notch filter (Leica, for RFP/Hoechst) and LAS X (Leica, version 3.5.5.19976) acquisition software under controlled environmental conditions (5% CO_2_, 37 °C).

For long term imaging, cerebral organoid chimeras grown in multi-well plates on microplate insert #3 were imaged with the TCS SP8 STED 3X microscope (Leica) under controlled environmental conditions (5% CO_2_, 37 °C). Confocal images of GFP expressing cells were taken every 20 min for 20.3 h with an HC PL APO CS2 20x/0.75 DRY objective lens (1024 × 1024 pixels at 0.568 μm/pixel). A 488 nm (20%) laser line transmission of a white light laser (50% power) was used for excitation. A Hybrid detector (HyD, gain: 100, offset: 0) was used for detection, with a gated range of 500–598 nm. A total of 141 Z slices were captured, each 0.685 μm apart. Of these, slices 66–100 were selected for maximum Z projection (LAS X) and further analysis, with the remaining slices excluded to improve visualisation of individual cellular movements.

To investigate RFP-expressing PD-GSCs within cerebral organoids and analyse the process of tumour cell invasion, channels were imaged sequentially, with a white light laser (50% power) used to excite RFP (PD-GSCs) using a 594 nm laser line (20% intensity), while a diode 405 laser was used for Hoechst 33342 (nuclei), with a 405 nm laser line (2% intensity). Hybrid detectors (HyDs, gain: 100, offset: 0) were used to collect emission within the ranges of 417–546 nm for Hoescht 33342 and 605–690 nm for RFP. Time lapse imaging was performed by acquisition every 30 mins for 17.5 h at 20X magnification and 0.75 zoom with an HC PL APO CS2 20x/0.75 DRY (Leica) objective lens (2048 × 2048 pixels at 0.379 μm/pixel). 63 Z slices 2.409 μm apart (total distance 150 μm) were taken for each time point and maximum Z projections were generated using the Leica LAS X image processing software (Leica).

To generate images of whole organoids, images were taken with an HC PL APO CS2 10x/0.40 DRY (Leica) objective lens using the LAS X Tile Scan acquisition mode. Four tiles (each 2048 × 2048 pixels at 0.757 μm/pixel) completed the total field of view, with 52 Z slices 5 μm apart (total distance 255 μm) taken for each tile. The LAS X software was used to generate maximum Z projections from the raw images for each tile and channel, and the tif files were exported. The single channel images were merged in FIJI, with no manipulation. The resulting 4 tiles were stitched into a single image (3536 × 3540 pixels, 0.757 μm /pixel) using the Image J plugin developed by Preibisch and collaborators [66], with the following settings distinct from default [Tile overlap: 27.7%; Fusion method: Intensity of random input tile; Compute overlap: active; Subpixel accuracy: active; Grid size: 2 × 2] for the most accurate alignment while minimising duplication of nuclei in the overlap region.

For figure preparation, all images were worked on their original resolutions and only altered their size for the purpose of preparation of figures to adjust to a resolution of 300 ppi in Adobe Illustrator.

### PD-GSC microinjection in brain organoids

For microinjection of RFP expressing PD-GSC into 30 day-old hESC-derived brain organoids, we used a Microinjection Syringe Pump/SMARTouch Controller (UMP3T-1, World Precision Instruments, USA) equipped with a glass 25 μL syringe and pulled pipette. For this, a suspension of cells was made in DPBS containing Brilliant Blue colorant. We used a 25 μL glass syringe (Hamilton) with a glass pulled pipette and a 30 μm tip (Fivephoton Biochemicals, USA). The cell concentration per injection was 9 × 10^3^ cells/μL (2 × 0.25 μL per organoid) and injections were performed at 3 nL/sec.

## Supplementary Information

**Additional file 1: Supplementary Figure 1** (related to Fig. 2). Representative sections of cerebral organoids grown on the micro-well inserts and stained with Hoechst 33342 and against cleaved caspase 3. Cleaved caspase 3 is observed in the periphery of cortical structures within organoids, without significant differences across the different experimental conditions (i.e. with or without microplate inserts). Panels were made with cropped images (same size for all time points and conditions). Image’s intensity levels were contracted (same extent for each of the channels across all conditions) from their original 16-bit range to 8-bit RGB for Figure preparation in Adobe Illustrator. Scale Bar = 100 μm.

**Additional file 2: Supplementary Movie 1** (related to Fig. 2)**.** Time-course imaging of brain organoids using different microplate inserts. Images were taken every 3.5 days for 50 days.

**Additional file 3: Supplementary Movie 2** (related to Fig. 4a)**.** Time course fluorescence microscopy imaging of chimera organoids over 20 h (images taken every 20 min time interval), showing neuronal progenitor cell behaviour.

**Additional file 4: Supplementary Movie 3** (related to Fig. 4d-e)**.** Time course fluorescence microscopy imaging of PD-GSC (grey) injected into hESC-derived brain organoids. At 48 h post-injection, Hoechst 33342 was added to the culture media for a 20 min incubation. Following a wash and media change, images were acquired every 30 min for 17.5 h.

**Additional file 5: Supplementary Table 1** (Related to Fig. 2). Linear regression results for measurements of organoid growth rates from brain organoids grown using different microplate inserts.

**Additional file 6: Supplementary Table 2** (Related to Fig. 3). RNA-seq results from brain organoids grown using different microplate inserts.

**Additional file 7: Supplementary Information.** This contains information related to 1) Image J script for image segmentation and quantification of different organoid parameters and 2) Matlab script for Spearman correlation coefficient analysis between RNA expression profiles.

## Data Availability

Computational scripts for image segmentation and RNA-seq analysis are provided in the Supplementary Information. Other data generated and analysed during this study are included in this published article [and its supplementary information files], except for microscopy image datasets, which are available from the corresponding author on reasonable request.

## Abbreviations

AD: Alzheimer disease
BSA: Bovine serum albumin
D-PBS: Dulbecco’s phosphate-buffered saline
EBs: Embryoid bodies
ESC: Embryonic stem cells
enCOR: Engineered cerebral organoids
GBM: Glioblastoma multiforme
GSC: Glioma stem cells
hESC: Human embryonic stem cells
iPSC: Induced pluripotent stem cells
NPCs: Neuronal progenitor cells
PD-GSC: Patient-derived glioma stem cells
RNA-seq: Bulk RNA sequencing
