# Supplementary figures and images for: 3D-printed microplate inserts for long term high-resolution imaging of live brain organoids
Source: BMC Biomed Eng. 2021 Apr 1;3:6. doi: 10.1186/s42490-021-00049-5 (PMC8015192; doi:10.1186/s42490-021-00049-5)

SUPPLEMENTARY FIGURE 1

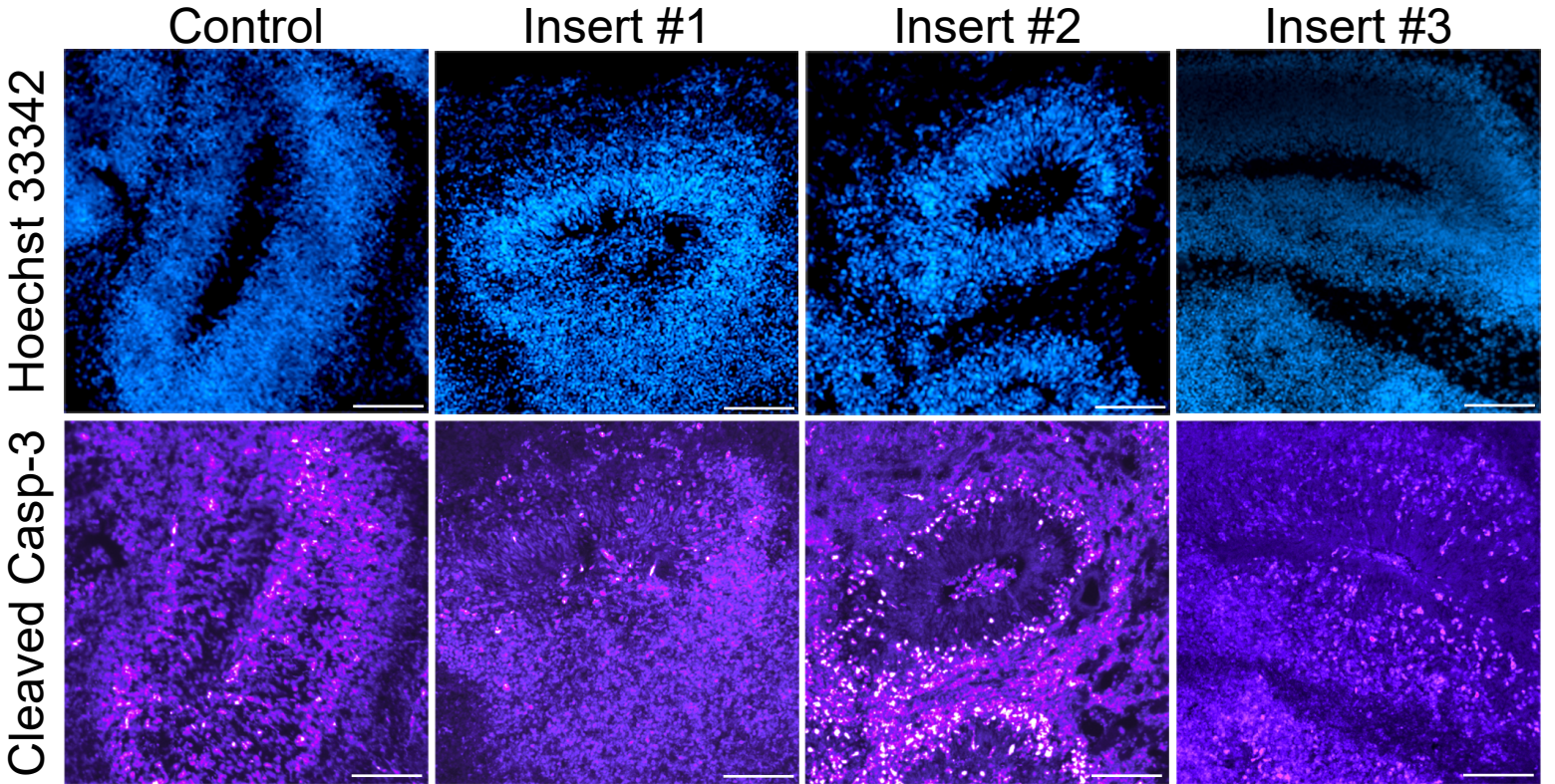

Supplement: Supplementary file 1 — Additional file 1: Supplementary Figure 1 (related to Fig. 2). Representative sections of cerebral organoids grown on the micro-well inserts and stained with Hoechst 33342 and against cleaved caspase 3. Cleaved caspase 3 is observed in the periphery of cortical structures within organoids, without significant differences across the different experimental conditions (i.e. with or without microplate inserts). Panels were made with cropped images (same size for all time points and conditions). Image’s intensity levels were contracted (same extent for each of the channels across all conditions) from their original 16-bit range to 8-bit RGB for Figure preparation in Adobe Illustrator. Scale Bar = 100 μm. [file 42490_2021_49_MOESM1_ESM.pdf]
