# Supplementary material for: 3D-printed microplate inserts for long term high-resolution imaging of live brain organoids
Source: BMC Biomed Eng. 2021 Apr 1;3:6. doi: 10.1186/s42490-021-00049-5 (PMC8015192; doi:10.1186/s42490-021-00049-5)
Supplement: Supplementary file 5 — Additional file 5: Supplementary Table 1 (Related to Fig. 2). Linear regression results for measurements of organoid growth rates from brain organoids grown using different microplate inserts. [file 42490_2021_49_MOESM5_ESM.pdf]

Inserts	Initial Organoid Size (mm)	p value	Growth Rate (mm /day)	p value
Control (Without Insert)	2.422± 0.7628	>0.9999	1.278± 2.849e-002	0.9978
Insert #1-flat cone	1.387± 0.7039	0.1935	1.307± 2.63e-002	0.323
Insert #2-grid cone	0.566 ± 0.9071	0.1037	1.41± 3.388e-002	0.0029
Insert #3-suspended grid	2.582 ± 0.5875	0.8322	1.144± 2.376e-002	<0.0001
