# Supplementary material for: 3D-printed microplate inserts for long term high-resolution imaging of live brain organoids
Source: BMC Biomed Eng. 2021 Apr 1;3:6. doi: 10.1186/s42490-021-00049-5 (PMC8015192; doi:10.1186/s42490-021-00049-5)
Supplement: Supplementary file 7 — Additional file 7: Supplementary Information. This contains information related to 1) Image J script for image segmentation and quantification of different organoid parameters and 2) Matlab script for Spearman correlation coefficient analysis between RNA expression profiles. [file 42490_2021_49_MOESM7_ESM.docx]

**SUPPLEMENTARY INFORMATION**

**Title Page**

**3D-printed microplate inserts for long term high-resolution imaging of live brain organoids.**

**Mariana Oksdath Mansilla^1*^, Camilo Salazar-Hernandez^1^, Sally L. Perrin^1^, Kaitlin G Scheer^1^, Gökhan Cildir^1^, John Toubia^1^, Kristyna Sedivakova^1^, Melinda N. Tea^1^, Sakthi Lenin^1^, Elise Ponthier^1^, Erica C.F. Yeo^1^, Vinay Tergaonkar^1,2,3^, Santosh Poonnoose^4,5^, Rebecca J. Ormsby^5^, Stuart M. Pitson^1,7^, Michael P. Brown^1,6,7^, Lisa M. Ebert^1^, and Guillermo A. Gomez^1*^.**

^1^Centre for Cancer Biology, SA Pathology and University of South Australia, Adelaide SA 5000, Australia. ^2^Institute of Molecular and Cell Biology (IMCB), Agency for Science, Technology and Research (A-STAR), Singapore, Singapore. ^3^Department of Pathology, Yong Loo Lin School of Medicine, National University of Singapore, Singapore. ^4^Department of Neurosurgery, Flinders Medical Centre, Adelaide, SA 5042, Australia. ^5^Flinders Health & Medical Research Institute, College of Medicine & Public Health, Flinders University, Adelaide SA 5042. ^6^Cancer Clinical Trials Unit, Royal Adelaide Hospital, Adelaide SA 5000, Australia. ^7^School of Medicine, University of Adelaide, Adelaide SA 5000, Australia.

*To whom correspondence should be addressed:

Dr. Mariana Oksdath Mansilla: [mariana.om8@gmail.com](mailto:mariana.om8@gmail.com)

Dr. Guillermo A. Gomez: [Guillermo.Gomez@unisa.edu.au](mailto:Guillermo.Gomez@unisa.edu.au)

**CONTENTS**

**1. Image J script for image segmentation and quantification of different organoid parameters.**

**2. Matlab script for Spearman correlation coefficient analysis between RNA expression profiles.**

**1. Image J script for image segmentation and quantification of different organoid parameters.**

Experiment_folder="G:/TAOF/Experimental data/InCell Analyzer 2200/2019-05/2019-05-21-Exp01/"

plate_folder="Experiment_01_Plate_02_Day25";

Number_of_wells=24;

Wells_per_row=6;

Wells_with_Organoids_in_ROW_A= newArray("1","6"); // need to specify

Wells_with_Organoids_in_ROW_B= newArray("1", "2", "3", "4", "5", "6"); // need to specify

Wells_with_Organoids_in_ROW_C= newArray("1", "2", "6"); // need to specify

Wells_with_Organoids_in_ROW_D= newArray(); // need to specify ("1", "2", "3", "4", "5", "6")

Channel_1_Formal_Name="(fld 1 wv FITC - FITC2)";

Channel_1_Short_Name="FITC"; //choose something short

Channel_2_Formal_Name="(fld 1 wv TL-Brightfield - Cy3)";

Channel_2_Short_Name="TL"; //choose something short

MeanFilter="radius=25 stack";

//////////////////////////////////////////////////////////////////////////////////////////////////////////////////////////////

run("Close All");

run("ROI Manager...");

selectWindow("ROI Manager");

run("Close");

Table.deleteRows(0, 10000);

Number_of_rows=Number_of_wells/Wells_per_row;

Total_Wells_in_ROW=Array.getSequence(Wells_per_row+1);

Total_Wells_in_ROW=Array.deleteValue(Total_Wells_in_ROW, 0);

plateID=Experiment_folder+plate_folder;

row_array= newArray("A", "B", "C", "D", "E", "F", "G", "H", "I", "J", "K", "L", "M", "N", "O", "P", "Q", "R", "S", "T", "U");

for (c=1; c<3; c++){

if (c==1){

channel=Channel_1_Formal_Name;

Channel_Short_Name=Channel_1_Short_Name;

}

if (c==2){

channel=Channel_2_Formal_Name;

Channel_Short_Name=Channel_2_Short_Name;

}

for (i = 0 ; i < Number_of_rows; i++) {

row=row_array[i];

///DEFINE ROW ARRAY

if (row=="A"){

Wells_with_Organoids_in_ROW=Wells_with_Organoids_in_ROW_A;

} else if (row=="B") {

Wells_with_Organoids_in_ROW=Wells_with_Organoids_in_ROW_B;

} else if (row=="C") {

Wells_with_Organoids_in_ROW=Wells_with_Organoids_in_ROW_C;

} else if (row=="D") {

Wells_with_Organoids_in_ROW=Wells_with_Organoids_in_ROW_D;

}

///////////////

Empty_Wells_in_ROW=ArrayDiff(Total_Wells_in_ROW, Wells_with_Organoids_in_ROW);

for (j=1; j <Total_Wells_in_ROW.length+1; j++) {

Well_name= row + Total_Wells_in_ROW [j-1];

Well_index=Total_Wells_in_ROW[j-1];

test_well= newArray(1);

test_well=Array.fill(test_well,Well_index);

test_with_organoids=ArrayUnion(test_well, Wells_with_Organoids_in_ROW);

Array.getStatistics(test_with_organoids, min, max, mean, stdDev);

value_with_organoids=max;

test_without_organoids=ArrayUnion(test_well, Empty_Wells_in_ROW);

Array.getStatistics(test_without_organoids, min, max, mean, stdDev);

if(value_with_organoids==Total_Wells_in_ROW [j-1]) {

well_folder=plate_folder+"_"+Well_name;

FileLocation=Experiment_folder+plate_folder+"/"+well_folder;

FileName=row+" - "+Total_Wells_in_ROW[j-1]+channel+".tif";

open(FileLocation+"/"+FileName);

} else {

newImage("Untitled", "16-bit black", 2048, 2048, 1);

run("Specify...", "width=1000 height=1000 x=512 y=512 centered");

run("Add...", "value=100000");

run("Select None");

}

rename(Well_name);

}

}

run("Images to Stack", "name="+plate_folder+"_"+Channel_Short_Name+ " use");

run("Stack to Hyperstack...", "order=xyczt(default) channels=6 slices=4 frames=1 display=Color");

run("Set Scale...", "distance=3076 known=1 pixel=1 unit=cm");

saveAs("Tiff", Experiment_folder+plate_folder+"_"+Channel_Short_Name+".tif");

if (c==1){

run("Duplicate...", "duplicate");

run("Threshold...");

setAutoThreshold("Otsu dark");

run("Convert to Mask", "method=Otsu background=Dark calculate");

run("Grays");

setAutoThreshold("Otsu dark");

run("Analyze Particles...", "size=0.0009-Infinity show=Masks display add in_situ stack");

Table.deleteRows(0, 23);

run("Fill Holes", "stack");

run("Grays");

saveAs("Tiff", Experiment_folder+plate_folder+"_OrgMask.tif");

imageCalculator("Multiply create stack", plate_folder+"_"+Channel_Short_Name+".tif",plate_folder+"_OrgMask.tif");

saveAs("Tiff", Experiment_folder+plate_folder+"_OrgMask.tif");

run("Set Measurements...", "area mean shape skewness stack display scientific redirect=None decimal=5");

run("Close All");

open(Experiment_folder+plate_folder+"_"+Channel_Short_Name+".tif");

roiManager("Measure");

run("Mean...", MeanFilter);

rename("Filtered");

open(Experiment_folder+plate_folder+"_"+Channel_Short_Name+".tif");

imageCalculator("Subtract create stack", plate_folder+"_"+Channel_Short_Name+".tif","Filtered");

close("Filtered");

close(plate_folder+"_"+Channel_Short_Name+".tif");

saveAs("Tiff", Experiment_folder+plate_folder+"_"+Channel_Short_Name+"_BS.tif");

roiManager("Measure");

roiManager("Save", Experiment_folder+plate_folder+"_OrgMask.zip");

close(plate_folder+"_"+Channel_Short_Name+"_BS.tif");

open(Experiment_folder+plate_folder+"_"+Channel_Short_Name+".tif");

}

}

// F U N C T I O N S .....................................................

function ArrayUnion(array1, array2) {

unionA = newArray();

for (i=0; i<array1.length; i++) {

for (j=0; j<array2.length; j++) {

if (array1[i] == array2[j]){

unionA = Array.concat(unionA, array1[i]);

}

}

}

return unionA;

}

// . . . . . . . . . . . . . . . . . . . . . . . . . . . . . . . . . . . .

function ArrayDiff(array1, array2) {

diffA= newArray();

unionA = newArray();

for (i=0; i<array1.length; i++) {

for (j=0; j<array2.length; j++) {

if (array1[i] == array2[j]){

unionA = Array.concat(unionA, array1[i]);

}

}

}

c = 0;

for (i=0; i<array1.length; i++) {

for (j=0; j<unionA.length; j++) {

if (array1[i] == unionA[j]){

c++;

}

}

if (c == 0) {

diffA = Array.concat(diffA, array1[i]);

}

c = 0;

}

for (i=0; i<array2.length; i++) {

for (j=0; j<unionA.length; j++) {

if (array2[i] == unionA[j]){

c++;

}

}

if (c == 0) {

diffA = Array.concat(diffA, array2[i]);

}

c = 0;

}

return diffA;

}

**2. Matlab script for Spearman correlation coefficient analysis between RNA expression profiles.**

%Organoid_data=[]

%Patient_data=[]

Organoid_conditions=15;

Patient_conditions=30;

Organoid_data(Organoid_data==0) = NaN;

Patient_data(Patient_data==0) = NaN;

clear('spearman','pvalues');

for OC=1:Organoid_conditions;

for PC=1:Patient_conditions;

[spearman(PC,OC),pvalues(PC,OC)] = corr(Organoid_data(:,OC),Patient_data(:,PC),'Type','Spearman','Rows','pairwise');

%[spearman2(PC,OC),pvalues(PC,OC)] = corr(Organoid_data(:,OC),Patient_data(:,PC),'Type','Spearman');

end

end

h = heatmap(spearman)
